# Supplementary material for: Effect of short-term prednisone on beta-cell function in subjects with type 2 diabetes mellitus and healthy subjects
Source: PLoS One. 2020 May 5;15(5):e0231190. doi: 10.1371/journal.pone.0231190 (PMC7199958; doi:10.1371/journal.pone.0231190)
Supplement: S1 File — (DOC) [file pone.0231190.s001.doc]

**
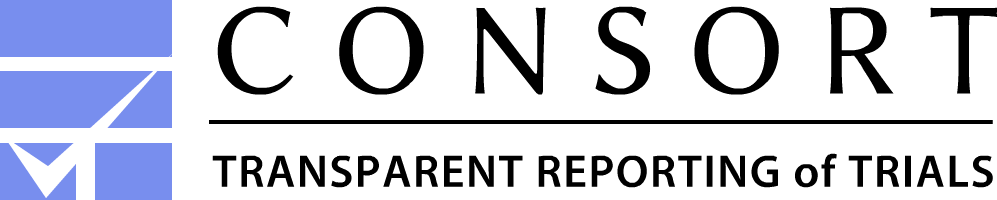
**

**CONSORT 2010 Flow Diagram**

**Allocation**

**Analysis**

**Follow-Up**

**Enrollment**

Assessed for eligibility (n= 10)

Excluded (n=0)

♦  Not meeting inclusion criteria (n=0)

♦  Declined to participate (n=0)

♦  Other reasons (n=0)

Analysed (n= 5)
♦ Excluded from analysis (give reasons) (n=0)

Lost to follow-up (give reasons) (n= 0)

Discontinued intervention (give reasons) (n= 0)

Allocated to intervention (n=5)

♦ Received allocated intervention (n=0)

♦ Did not receive allocated intervention (give reasons) (n=0)

Lost to follow-up (give reasons) (n= 0)

Discontinued intervention (give reasons) (n= 0)

Allocated to intervention (n=5)

♦ Received allocated intervention (n=0)

♦ Did not receive allocated intervention (give reasons) (n=0)

Analysed (n= 5)
♦ Excluded from analysis (give reasons) (n=0)

Allocated (n=10)
