# Supplementary material for: Effect of short-term prednisone on beta-cell function in subjects with type 2 diabetes mellitus and healthy subjects
Source: PLoS One. 2020 May 5;15(5):e0231190. doi: 10.1371/journal.pone.0231190 (PMC7199958; doi:10.1371/journal.pone.0231190)
Supplement: S2 File — (DOCX) [file pone.0231190.s002.docx]

**Research Proposal**

**Background**

We propose a trial to evaluate the effect of high-dose glucocorticoids on beta cell function in patients with a diseased pancreas, specifically type 2 diabetics.

Glucocorticoids are widely used in acute situations such as COPD exacerbations as well as chronic conditions such as rheumatoid arthritis, systemic lupus erythematosus or immune thrombocytopenic purpura. Many of these patients may have concurrent type 2 diabetes. More than half of patients who receive high dose glucocorticoids experience hyperglycemia whether or not they have diabetes (1). Management of these conditions is challenging due to a wide fluctuation in post-prandial hyperglycemia and the lack of clearly defined treatment protocols (2). Basal-bolus insulin is the current mainstay of treatment. Glucocorticoids cause hyperglycemia through impairment of multiple pathways resulting in beta cell dysfunction and insulin resistance in other tissues. Still, most of what we know regarding these mechanisms is from studies done on animal models or normal, healthy humans. A study looking at the effect of glucocorticoids on beta cell function in patients with type 2 diabetes has not been done before. Therefore, it is not known whether the changes in beta cell function seen in healthy subjects can be applied to this population. The purpose of this study is to look at the effect on beta cell function in terms of metabolic changes in glucose, insulin and C-peptide levels in diabetic patients exposed to high-dose glucocorticoids. The oral glucose tolerance test (OGTT) will be used to evaluate the parameters of beta cell function as it was demonstrated in a study done by Van Raalte, et al. that changes in fasting glucose levels were mild compared to the changes in postprandial glucose levels in healthy Caucasian men exposed to prednisolone treatment. This study implied that in order to study the effects of glucocorticoids on beta cell function and glucose metabolism, measurements should be done under stimulated conditions such as with an OGTT (3).

Resources:

1. Donihi AC, Raval D, et al. Prevalence and predictors of corticosteroid-related hyperglycemia in hospitalized patients. Endocr Pract 2006; 12: 358-362.
2. Hwang JL and Weiss RE. Steroid-induced diabetes: a clinical and molecular approach to understanding and treatment**.** Diabetes Metab Res Rev 2014; 30: 96-102.
3. Van Raalte DH, Brands M, et al. Low-dose glucocorticoid treatment affects multiple aspects of intermediary metabolism in healthy humans: a randomized controlled trial. Diabetologia 2011; 54: 2103-2112.

**Study Protocol**

A total of 5 patients with type 2 diabetes followed in the diabetes center will be eligible to be included in this study. After being checked into the diabetes clinic, subjects with type 2 diabetes will be screened for the study. Each day one of the study staff will review the list of patients being seen in the diabetes clinic in the electronic medical record (Cerner). If the patient meets all entry criteria and has no exclusion criteria, study staff will approach the potential subject. Any additional questions will be asked if needed to assess eligibility. After explaining all aspects of the study to the patient, the patient will be given the opportunity to ask any questions and to think about their decision/discuss with family for as much time as they need. After giving informed consent, subjects will be scheduled to return to the clinic for the three-day study protocol.

For a period of three days prior the study protocol, participants will be asked to check blood glucose (BG) levels twice daily – fasting and before either lunch or dinner – and to record these values. On their scheduled day, participants will be instructed to come to the clinic after an overnight fast of a minimum of 8 hours. They will be asked to refrain from drinking alcohol for a period of 24 hours before the study days and to not perform strenuous exercise for a period of 48 hours before the study days. They will be directed to avoid alcohol and strenuous exercise for the duration of the study. Participants on an NPH/regular insulin regimen will be instructed to not take their AM insulin on the first and last days of the study period. They will be asked to bring their insulin as well as food with them. After the OGTT, they will be advised to eat and take their insulin. Subjects on oral anti-diabetes medication or glargine can continue their medications as directed by their physician.

On days 1 and 3 of the study period, participants will be checked into the clinic. A physical examination, including height, weight and waist circumference will be performed. A 75-g OGTT will be performed with venous samples for measurement of glucose, insulin and C-peptide levels obtained at 0, 30 and 60 minutes starting immediately after the ingestion of the 75 g glucose solution. After samples are collected on day 1, participants will take their first dose of steroid, prednisone 40 mg, and be given a packet of two additional doses for the rest of the study period. Subjects will take prednisone 40 mg once daily for three days to be consumed around 8:00am on days 2 and 3. This dose was chosen as a typical high-dose glucocorticoid regimen. On the third day, subjects will again visit the clinic after an overnight fast of a minimum 8 hours and undergo a physical examination and OGTT just as on day 1.

On days 1-5 of the study period, participants will be instructed to check fasting (around 8:00 am), pre-prandial, and bed time (around 10:00 pm) BG levels and to record all values. A member of the study staff will call each participant daily around 7:00 pm to obtain BG values. Compliance with the study protocol will be assessed at this time as well. Any subject not compliant with study medications will be excluded from the study.

**Study Population**

**Inclusion Criteria:**

1. Males or non-pregnant females between the ages of 18-35 years being followed in the Diabetes Center.
2. **Type 2 Diabetes for ≤ 5 years and treated with any combination of metformin, saxagliptin, sitagliptin, linagliptin, alogliptin, repaglinide, nateglinide, pioglitazone, exenatide, liraglutide, or a total daily dose of insulin ≤ 0.4 units/kg/day.**
3. BMI 22.0-35.0 kg/ m^2^
4. HBA1c ≤ 8.0%

**Exclusion Criteria:**

1. Patients on sulfonylurea therapy (i.e. glimepiride, glyburide, or glipizide).
2. Patients with impaired renal function (estimated GFR less than 60 ml/min based on results from Cerner).
3. Patient who have received glucocorticoid therapy within six months of study.
4. Patients who do shift work.
5. Blood glucose level ≥ 300 mg/dl at clinic visit.
6. Signs or symptoms of infection.

**Safety/Treatment of Hyperglycemia**

Participants will be instructed to measure fasting, pre-prandial, and bedtime BG levels. In order to ensure the safety of the participants, study coordinators will call them daily on days 1-5 to obtain BG values. Subjects will be advised to come to the clinic if BG reading is ≥ 400 mg/dl or to the emergency room (ER) if after normal business hours. Participants seen in clinic will be reassessed by a study coordinator and a physician. BG and point of care (POC) urinalysis for ketones will be checked and the clinical status of the patient will be evaluated. It will be determined whether continuation in the study or discontinuation of participation is warranted. If BG is ≥ 300 mg/dl and < 400 mg/dl, participants will be advised to check their urine for ketones with urine Ketostix provided to them on the first day of the study protocol. If urine ketones are positive, they will be advised to go the ER. If negative, they will be seen the next day in the diabetes center and evaluated as above. If BG is < 300 mg/dl, no changes in medication or insulin dosing will be made due to the transitory nature of this intervention and the risk of nocturnal hypoglycemia once the glucocorticoid effect wears off.

Participants will be counseled before the study to expect hyperglycemia and that this will improve after the glucocorticoids are stopped. They will be advised to not make any changes in insulin dosing without consulting study coordinators. Participants will be given contact information of study coordinators and instructed to call if any questions or concerns arise at any time during the study period.

**Study Outcomes**

The primary study outcome of the study is:

1. The difference in the area under the curve of c-peptide (AUC_CP_) after OGTT at the beginning and end of the study.

The secondary study outcomes will include:

1. The difference in the area under the curve of glucose (AUC_G_) after OGTT at the beginning and end of the study.
2. The difference in fasting AM glucose level at the beginning and end of the study.
3. The difference in fasting c-peptide level at the beginning and end of the study.
4. The difference in HOMA-B and HOMA-IR at the beginning and end of the study.
5. The percentage of patients who became hyperglycemic during the study.
6. The time it took for those participants to become hyperglycemic.
7. The time it took for those participants to return to pre-glucocorticoid BG levels.

**Data Analysis**

The homeostasis model assessment of B-cell function (HOMA-B) and homeostasis model assessment of insulin resistance (HOMA-IR) will be calculated as described by Matthews et al. HOMA-B and HOMA-IR with use of C-peptide values will be calculated as described by Li et al for patients with diabetes. The B-cell response to the OGTT will also be calculated as area under the curve (AUC) for C-peptide at 0, 30, and 60 minutes by using the trapezoidal rule.
