# Supplementary material for: Effect of short-term prednisone on beta-cell function in subjects with type 2 diabetes mellitus and healthy subjects
Source: PLoS One. 2020 May 5;15(5):e0231190. doi: 10.1371/journal.pone.0231190 (PMC7199958; doi:10.1371/journal.pone.0231190)
Supplement: S3 File — (DOC) [file pone.0231190.s003.doc]

**­­­­­­­­­­IRB #_16-035_____________**

**Submission for CCHHS IRB APPROVAL ­ ______________________**

**Date**

**III. a INVESTIGATORS' ACKNOWLEDGMENT**

**Complete for each investigator taking scientific responsibility for this project**

1. Yannis Guerra, MD

Typed name Responsible Investigator (Principal County Investigator)

**_­­­_____________________________________________**date_____________________________

**Signature indicates: I have completed the CCHHS human subjects research training and**

**I understand that no changes may take place without prior IRB review and approval.**

**Financial interest in this research (circle). NO YES (IF YES, complete, Financial Interest Statement, form II)**

Attending Physician, Endocrinology, Internal Medicine 1900 W. Polk St. Room 811

Appointment, Department, Division address room

(312) 864-0522 333-8853 (312) 864-9735 yguerra@cookcountyhhs.org

Phone Page Fax Email address

2. Leon Fogelfeld, MD

Typed name of Co-PI

_______________________________________________date____________________________

**Signature indicates: I have completed the CCHHS human subjects research training.**

**Financial interest in this research (circle). NO YES (IF YES, complete, Financial Interest Statement, form II)**

Chair, Endocrinology, Internal Medicine 1900 W. Polk St. Room 811

Appointment, Department, Division address

(312) 864-0539 (312) 864-9735 lfogelfeld@cookcountyhhs.org

Phone Fax Email address

**Use additional pages as necessary to list all persons responsible for this research.**

3. Monica Shah, MD

Typed name of Investigator

_____________________________________________date_________­­­­­­­___________________

**Signature indicates: I have completed the CCHHS human subjects research training.**

**Financial interest in this research (circle). NO YES (IF YES, complete, Financial Interest Statement, form II)**

Fellow Physician, Internal Medicine, Endocrinology 1900 W. Polk St. Room 801

Appointment, Department, Division address

(312) 864 -0544 (312) 864-9735 Monica_Shah@rush.edu

Phone Fax Email address

4. May Adel, MD

Typed name of Investigator

______________________________________________date____________________________

**Signature indicates: I have completed the CCHHS human subjects research training.**

**Financial interest in this research (circle). NO YES (IF YES, complete, Financial Interest Statement, form II)**

Research Coordinator, Internal Medicine, Endocrinology 1900 W. Polk St. Room 809

Appointment, Department, Division address

(312) 250 -0942 (312) 864-8890 malassadi@cookcountyhhs.org

Pager Fax Email address

5.

Typed name of Investigator

**_______________________________________________**date__________________________

**Signature indicates: I have completed the CCHHS human subjects research training.**

**Financial interest in this research (circle). NO YES (IF YES, complete, Financial Interest Statement, form D-II)**

Appointment, Department, Division address

Phone Fax Email address

**III. b ABSTRACT OF RESEARCH PROPOSAL**

# Title: The Effect of Glucocorticoids on Beta Cell Function in Type 2 Diabetes

We propose a trial to evaluate the effect of high-dose glucocorticoids on beta cell function in patients with a diseased pancreas, specifically type 2 diabetics, compared to healthy controls.

Glucocorticoids are widely used in acute conditions such as COPD exacerbations as well as chronic disease processes such as rheumatoid arthritis, systemic lupus erythematosus or immune thrombocytopenic purpura. Many of these patients may have concurrent type 2 diabetes. More than half of patients who receive high dose glucocorticoids experience hyperglycemia whether or not they have diabetes (1). Management of these conditions is challenging due to a wide fluctuation in postprandial hyperglycemia and the lack of clearly defined treatment protocols (2). Basal-bolus insulin is the current mainstay of treatment. Glucocorticoids cause hyperglycemia through impairment of multiple pathways resulting in beta cell dysfunction and insulin resistance in other tissues. Still, most of what we know regarding these mechanisms is from studies done on animal models or normal, healthy humans. A study looking at the effect of glucocorticoids on beta cell function in patients with type 2 diabetes has not been done before. Therefore, it is not known whether the changes in beta cell function seen in healthy subjects can be applied to this population. The purpose of this study is to look at the effect on beta cell function in terms of metabolic changes in glucose, insulin and C-peptide levels in diabetic patients exposed to high-dose glucocorticoids. The oral glucose tolerance test (OGTT) will be used to evaluate the parameters of beta cell function as it was demonstrated in a study done by Van Raalte, et al. that changes in fasting glucose levels were mild compared to the changes in postprandial glucose levels in healthy Caucasian men exposed to prednisolone treatment. This study implied that in order to study the effects of glucocorticoids on beta cell function and glucose metabolism, measurements should be done under stimulated conditions such as with an OGTT (3).

Resources:

1. Donihi AC, Raval D, et al. Prevalence and predictors of corticosteroid-related hyperglycemia in hospitalized patients. Endocr Pract 2006; 12: 358-362.
2. Hwang JL and Weiss RE. Steroid-induced diabetes: a clinical and molecular approach to understanding and treatment**.** Diabetes Metab Res Rev 2014; 30: 96-102.
3. Van Raalte DH, Brands M, et al. Low-dose glucocorticoid treatment affects multiple aspects of intermediary metabolism in healthy humans: a randomized controlled trial. Diabetologia 2011; 54: 2103-2112.

**Sponsor and/or funding source: _____**Self-funded**__________________**

| **Indicate whether Funds have been *committed* or whether this is in the Proposal phase. F P**  **Does this research involve an investigational drug or device?** ______ No**_____** |
| --- |

**Study objective:** To evaluate the effect of high-dose glucocorticoids on beta cell function in patients with a diseased pancreas, specifically type 2 diabetics, compared to healthy controls.

.

**Recruitment Procedure**:

A total of 10 patients (5 with type 2 diabetes and 5 healthy controls) will be eligible to be included in this study. The diabetic patients will be recruited from the diabetes center at the Fantus clinic. Each day one of the study staff will review the list of patients being seen in the diabetes clinic in the electronic medical record (Cerner). If the patient meets all entry criteria and has no exclusion criteria, study staff will approach the potential subject. Any additional questions will be asked if needed to assess eligibility. After explaining all aspects of the study to the patient, the patient will be given the opportunity to ask any questions and to think about their decision/discuss with family for as much time as they need. After giving informed consent, subjects will be scheduled to return to the clinic for the three-day study protocol. The control subjects will be recruited through advertisements. Potential study subjects will call one of the study coordinators and if they meet criteria based on history, they will be scheduled to come to the clinic for the three-day study protocol. At their scheduled visit, weight and fasting plasma glucose will be checked to ensure they meet eligibility prior to oral glucose tolerance testing.

**Please describe your plan to protect patient confidentiality and to secure data during the screening and all other phases of the study.**

Are you sending, sharing, storing or allowing access of Protected Health Information off campus, or to any CCHHS party who would otherwise NOT have access? Y N

We will remove patient's identifiers after data collection phase. We will store all PHI data on access restricted folders in the CCHS intranet. We will not share information with outside CCHS parties.

**Design/Intervention**:

For a period of two days prior the study protocol, participants will be asked to check blood glucose (BG) levels twice daily – fasting and before either lunch or dinner – and to record these values. On their scheduled day, participants will be instructed to come to the clinic after an overnight fast of a minimum of 8 hours (subjects can drink water). They will be asked to refrain from drinking alcohol for a period of 24 hours before the study days and to not perform strenuous exercise for a period of 48 hours before the study days. They will be directed to avoid alcohol and strenuous exercise for the duration of the study.

On days 1 and 3 of the study period, participants will be checked into the clinic. A physical examination, including height, weight and waist circumference, and finger-stick glucose will be performed. A 75-g OGTT will be performed with venous samples for measurement of glucose, insulin and C-peptide levels obtained at 0, 30 and 60 minutes starting immediately after the ingestion of the 75 g glucose solution. Samples for basic metabolic profile and liver function tests will also be collected at time 0. After samples are collected on day 1, participants will be administered their first dose of steroid, prednisone 40 mg, and be given a packet of two additional doses for the rest of the study period. We may only give 1 additional dose to them and have them take the last dose in clinic on day 3 two hours before the OGTT. Subjects will take prednisone 40 mg once daily for three days to be consumed around 8:00am on days 2 and 3. This dose was chosen as a typical high-dose glucocorticoid regimen. On the third day, subjects will again visit the clinic after an overnight fast of a minimum 8 hours and undergo a physical examination and OGTT just as on day 1.

Participants will be instructed to check pre-prandial (before breakfast, lunch and dinner), and bed time (around 10:00 pm) BG levels and to record all values for a total of five days (three days while on prednisone and for two days after the second study visit). A member of the study staff will call each participant daily around 7:00 pm to obtain BG values. Compliance with the study protocol will be assessed at this time as well. Any subject not compliant with study medications will be excluded from the study.

Total number of subjects everywhere: ___10____ At County____10____

Inclusion/exclusion criteria:

Experimental Group

Inclusion Criteria:

1. Males or non-pregnant females between the ages of 18-50 years being followed in the Diabetes Center.
2. Type 2 Diabetes for ≤ 1 year and only on metformin
3. BMI 24.0-35.0 kg/ m^2^
4. HBA1c ≤ 9.0%

Exclusion Criteria:

1. Patients on any other antidiabetes medication.
2. Patients with impaired renal function (estimated GFR less than 60 ml/min based on results from Cerner).
3. Patient who have received glucocorticoid therapy within six months of study.
4. Patients who do shift work.
5. Blood glucose level ≥ 250 mg/dl on finger-stick at clinic visit.
6. Signs or symptoms of infection.
7. Patients with a history of pancreatitis.
8. Patients with a history of drug or alcohol abuse.

Control Group

Inclusion Criteria:

1. Males or females between the ages of 18-50 years in good physical health as determined by medical history, physical examination, and screening blood tests
2. Normoglycemia defined by fasting plasma glucose < 5.6 mmol/L (or 100 mg/dL) and 2h glucose <7.8 mmol/L (or 140 mg/dL) after a 75 g OGTT
3. BMI: 22.0 – 28.0 kg/m^2^

Exclusion Criteria:

1. Presence of any disease or use of any medication
2. Patients with a first-degree relative with Type 2 Diabetes
3. Patients who smoke
4. Patients with history of steroid use
5. Patients who do shift work
6. Patients with recent changes in weight or physical activity

End points: (Outcomes to be measured)

The primary study outcome of the study is:

1. The difference in the area under the curve of c-peptide (AUC_CP_) after OGTT at the beginning and end of the study.

The secondary study outcomes will include:

1. The difference in the area under the curve of glucose (AUC_G_) after OGTT at the beginning and end of the study.
2. The difference in fasting AM glucose level at the beginning and end of the study.
3. The difference in fasting c-peptide level at the beginning and end of the study.
4. The difference in HOMA-B and HOMA-IR at the beginning and end of the study.
5. The percentage of patients who became hyperglycemic during the study.
6. The time it took for those participants to become hyperglycemic.
7. The time it took for those participants to return to pre-glucocorticoid BG levels.

Resource:

1. Umpierrez GE, Gianchandani R, et al. Safety and efficacy of sitagliptin therapy for the inpatient management of general medicine and surgery patients with type 2 diabetes: a pilot, randomized, controlled study. Diabetes Care 2013; 11: 3430 – 3435.

Will interim data and safety monitoring be carried out during this study? No______

**Safety:**

Participants will be instructed to measure pre-prandial and bedtime BG levels. In order to ensure the safety of the participants, study coordinators will call them daily on days 1-5 to obtain BG values. Subjects will be advised to come to the clinic if BG reading is ≥ 400 mg/dl or to the emergency room (ER) if after normal business hours. Participants seen in clinic will be reassessed by a study coordinator and a physician. BG and point of care (POC) urinalysis for ketones will be checked and the clinical status of the patient will be evaluated. The need for additional antidiabetes medication will be assessed and it will be determined whether continuation in the study or discontinuation of participation is warranted. If BG is ≥ 300 mg/dl and < 400 mg/dl, participants will be advised to check their urine for ketones with urine Ketostix provided to them on the first day of the study protocol. If urine ketones are positive, they will be advised to go the ER. If negative, they will be seen the next day in the diabetes center and evaluated as above. If BG is < 300 mg/dl, no changes in medication dosing will be made due to the transitory nature of this intervention and the risk of nocturnal hypoglycemia once the glucocorticoid effect wears off.

Participants will be counseled before the study to expect hyperglycemia and that this will improve after the glucocorticoids are stopped. They will be advised to not make any changes in medication dosing without consulting study coordinators. Participants will be given contact information of study coordinators and instructed to call if any questions or concerns arise at any time during the study period. If blood glucose levels do not return to normal two days after the second study visit, a follow-up appointment will be made in the outpatient diabetes clinic.

Power calculation or rationale for sample size:

The sample size of 5 patients was estimated on the basis of an expected standard deviation difference of 0.55, an α of 0.05, and a power of 80%. These estimates were based on the area under the curve for c-peptide values from a recent study investigating B-cell function in type 2 diabetics after liraglutide treatment (1).

Resource:

1. Kondo Y, Satoh S, et al. Early liraglutide treatment improves B-cell function in patients with type 2 diabetes: a retrospective cohort study. Endocrine Journal 2015; 62: 971-980.

**List Fellows, Residents or Students who will be participating in this project:**

| **Name** | **Dept/Div** | **Fellow** | **Resident** | **Student** |
| --- | --- | --- | --- | --- |
| Monica Shah, MD | Internal Medicine/  Endocrinology | X |  |  |
|  |  |  |  |  |
|  |  |  |  |  |

Has each completed the human subject research self-guided tutorial? Y N

**III. Description of Subjects: (Indicate NUMBER for each category, leave no blanks)**

**If study changes to include subjects not indicated, a revision must be approved prior to inclusion**

**# of Competent Adults………………………………………………………………___10_________**

**# of Pregnant Women (If included, complete D-IVWS)…………………………..___0________**

**# of Decisionally-impaired or unconscious Adults**

**including persons in severe pain or receiving pain medication………………..­­­­...____0________**

**(Surrogate Consent for legal representative to be used must be submitted)**

**# of Employees……………………………………………………………………… ___5________**

**# of Minors (what age?)…………………………………………………………….____0________**

**Please indicate:**

**If yes, Is this study minimal risk? 45CFR46.404 Y__ N__**

**Does this study hold a prospect of benefit to each subject? 45CFR46.405 Y__ N__**

**Or, is this study for the benefit of the community? 45CFR46.406 ___________Y__ N__**

**# of Wards of the State or Foster Children (complete-WS)……………………_____0________**

**(CCHHS does not permit wards in 45CFR46.406 research)**

**# of Detainees or identified records of detainees (complete DS)…..…………. _____0________**

**(persons on parole should be included)**

**What steps are taken to avoid coercion of vulnerable subjects:**

We are not using any subject in this category due to the characteristics of our study

Approval: IRB # ______________________

**Signature of IRB Chair or Designee Date:**

Remarks:
