# Supplementary material for: Effect of short-term prednisone on beta-cell function in subjects with type 2 diabetes mellitus and healthy subjects
Source: PLoS One. 2020 May 5;15(5):e0231190. doi: 10.1371/journal.pone.0231190 (PMC7199958; doi:10.1371/journal.pone.0231190)
Supplement: Attachment — Submitted filename: Response to reviewers 2-13-20.docx [file pone.0231190.s006.docx]

Reviewers' comments:

Reviewer's Responses to Questions

**Comments to the Author**

1. Is the manuscript technically sound, and do the data support the conclusions?

The manuscript must describe a technically sound piece of scientific research with data that supports the conclusions. Experiments must have been conducted rigorously, with appropriate controls, replication, and sample sizes. The conclusions must be drawn appropriately based on the data presented.

Reviewer #1: No

Reviewer #2: Yes

Reviewer #3: Yes

2. Has the statistical analysis been performed appropriately and rigorously?

Reviewer #1: No

Reviewer #2: Yes

Reviewer #3: Yes

3. Have the authors made all data underlying the findings in their manuscript fully available?

The [PLOS Data policy](https://gcc01.safelinks.protection.outlook.com/?url=http%3A%2F%2Fwww.plosone.org%2Fstatic%2Fpolicies.action%23sharing&data=02%7C01%7Cyguerra%40cookcountyhhs.org%7C74cbdfb597bb4f78587008d78dee6e59%7C3b922295e886417faaa84e4c4f069d82%7C0%7C1%7C637133927514874831&sdata=e3wZxHsSGrERMlAcl%2FVnNe3OcBgpnQhceWjz9kWHFLs%3D&reserved=0) requires authors to make all data underlying the findings described in their manuscript fully available without restriction, with rare exception (please refer to the Data Availability Statement in the manuscript PDF file). The data should be provided as part of the manuscript or its supporting information, or deposited to a public repository. For example, in addition to summary statistics, the data points behind means, medians and variance measures should be available. If there are restrictions on publicly sharing data—e.g. participant privacy or use of data from a third party—those must be specified.

Reviewer #1: Yes

Reviewer #2: Yes

Reviewer #3: Yes

4. Is the manuscript presented in an intelligible fashion and written in standard English?

PLOS ONE does not copyedit accepted manuscripts, so the language in submitted articles must be clear, correct, and unambiguous. Any typographical or grammatical errors should be corrected at revision, so please note any specific errors here.

Reviewer #1: Yes

Reviewer #2: Yes

Reviewer #3: Yes

5. Review Comments to the Author

Please use the space provided to explain your answers to the questions above. You may also include additional comments for the author, including concerns about dual publication, research ethics, or publication ethics. (Please upload your review as an attachment if it exceeds 20,000 characters)

Reviewer #1: Although the authors addressed a clinical issue about the effect of prednisone on beta-cell function between T2DM patients and healthy people, the sample size was too small to support the conclusion, and the mismatch of age and gender lead to large sample bias. To the reviewer's opinion, more rigorous experiments and data were needed to confirm the conclusions.

*We agree with the concerns of Reviewer #1. The size used was based on our sample calculation for the limited outcomes measured. Despite these concerns there were significant differences between the two groups. To avoid type 1 error, there is a need to confirm the study findings with bigger and better matched groups. We indicated this in the limitations (page 8, line 159-161) and in the conclusion (page 9, line 172).*

Reviewer #2: Shah and colleagues drew a preliminary but interesting study concluding high-dose prednisone resulting in suppressed insulin secretion rather than increasing insulin resistance. The conclusion seems result-based, but some concerns should be noted before its publication.

1. How did the author confirm the diagnosis of type 2 diabetes? As this is a very small sample trial, the representation of the study is exclusively important (authors may wish to add differentiating information like auto-antibodies, baseline 8 am cortisol and other MODY phenotypes).

*We confirmed the diagnosis of type 2 diabetes using the standard criteria of A1c above 6.5% (present in 5/5 cases and 0/5 controls). In addition, our patients underwent OGTT, which showed criteria for diagnosis of DM2 in 4/5 cases and 0/5 controls. All patients had normal C-peptide production levels at baseline time for OGTT, which would rule out significant autoimmune diabetes as the cause of hyperglycemia. MODY phenotypes were not likely since those with diabetes were relatively well-controlled and none were treated with sulfonylurea medications.*

2. The therapeutic regimens need to be provided including a daily dose of metformin. If there is any dose change of metformin during the trial, it should be reported.

*Of the five patients with diabetes, three were treated with metformin before and during the study and two were without diabetes medications (added to pages 5-6, lines 105-107); there was no change to any doses of medications. Patients were informed of the importance of continuing all their medications during the time of the prednisone administration.*

3. Are there any other safety issues reported in the trial, such as hypertension, insomnia, etc?

*No other safety issues were reported per patient after interview with research assistant on day of 3^rd^ dose administration.*

4. The reduction of AUC C-peptide and AUC Insulin is not totally in line. Please explain.

*The reduction of AUC of both C-peptide and insulin were in the same direction for the group of subjects with diabetes. For the healthy subjects, the explanation for discordant direction of the insulin and C-peptide may lie in the outlier values of two patients in whom both the insulin and C-peptide went up but only minimally.*

5. The authors may wish to provide some clinical interpretations in steroid usage, especially the clinical context of a three-day prescription of 40mg/d prednisone.

*The dose selected was similar to that used in study of healthy volunteers. This is also the dose recommended for short courses of steroids in case of COPD or asthma exacerbation. This explanation is included in the introduction (page 3, lines 44-45).*

6. The authors may also wish to discuss the underlying pathophysiological mechanism of the study result in more detail.

*Discussion of the mechanisms causing the beta cell impairment was amplified (page 8, lines 145-149).*

7. This trial does not include a dose gradient of prednisone, which is also a limitation needing stated.

*The few data available show that dose ranges from 30-75 mg/day do not seem to cause different effects in patients without diabetes mellitus. See page 3, lines 42-45 for rationale of dosing.*

8. I think "emergent response of beta-cell function" rather than "short-short effects" may more fit the study context.

*Wording was changed on page 9, line 162 to incorporate this suggestion.*

9. Please note some typos like OGGT in the manuscript.

*Corrections made.*

Reviewer #3: In their manuscript entitled “Effect of short-term prednisone on beta-cell function in subjects with type 2 diabetes mellitus and healthy subjects," Shah and colleagues report that that a short course of high-dose glucocorticoids in those with T2DM leads to a significant decrease in insulin secretion. However, the healthy controls did not have significant decreases in beta-cell insulin secretion parameters after prednisone exposures. The authors believe that subjects with T2DM have an increased beta-cell impairment and reduced ability to recover after glucocorticoid exposure.
Overall, the authors report very interesting results; however, a few issues do require attention in my opinion.

1. In fact, many studies have demonstrated that glucocorticoids treatment may lead to insulin resistance, increased hepatic gluconeogenesis and hyperglycemia in animals and human. Of note, these effects of glucocorticoids are dependent on long term treatment (for example, 2 weeks or 3 weeks). However, in the present study, human subjects were treated with glucocorticoid for 3 days. Thus, the authors just observed short effects of glucocorticoid treatment. These data are interesting. However, when the authors make conclusion, they should be more objective and fair. In my opinion, they should conclude that short-term glucocorticoid treatment may impair insulin secretion and beta cell in patients with type 2 diabetes. They cannot deny the long-term effects of glucocorticoids on insulin resistance and hepatic gluconeogenesis.

*We agree with reviewer, that the long term effect of the glucocorticoids in healthy volunteers has been shown multiple times. Our study is the first to evaluate the response in patients with type 2 diabetes, although only for the initial 72 hours of administration. We agree that studies still need to be done for effects over the long term. Edits have been made on page 9, lines 170-172 to reflect this.*

2. The mechanism of this study is completely unclear, and the mechanism of how glucocorticoid affects the decrease of insulin secretion by beta cells in diabetic patients is unclear. The authors should discuss this issue, based on the previous literatures

*Discussion of the mechanisms causing the beta cell impairment was amplified (page 8, lines 145-149).*

3. The number of participants is not enough. The author also mentioned this point and acknowledged this weakness.

*The size used was based on our sample calculation for the limited outcomes measured. Despite these concerns there were significant differences between the two groups. To avoid type 1 error, there is a need to confirm the study findings with bigger and better matched groups. We indicated this in the limitations (page 8, line 159-161) and in the conclusion (page 9, line 172).*

4. What were the results in animals? Has the authors performed similar experiments in mice?

*We are exclusively a clinical research site. Data on animals has been done before (Lambillote et al). We had hoped to show the effect of these drugs on a population that has not been studied directly before (humans with type 2 diabetes).*

6. PLOS authors have the option to publish the peer review history of their article ([what does this mean?](https://gcc01.safelinks.protection.outlook.com/?url=https%3A%2F%2Fjournals.plos.org%2Fplosone%2Fs%2Feditorial-and-peer-review-process%23loc-peer-review-history&data=02%7C01%7Cyguerra%40cookcountyhhs.org%7C74cbdfb597bb4f78587008d78dee6e59%7C3b922295e886417faaa84e4c4f069d82%7C0%7C1%7C637133927514874831&sdata=00uTGP9vCJuU1KMsKdibdRAwEBpDgh98nJdOZYIuMYk%3D&reserved=0)). If published, this will include your full peer review and any attached files.


If you choose “no”, your identity will remain anonymous but your review may still be made public.


**Do you want your identity to be public for this peer review?** For information about this choice, including consent withdrawal, please see our [Privacy Policy](https://gcc01.safelinks.protection.outlook.com/?url=https%3A%2F%2Fwww.plos.org%2Fprivacy-policy&data=02%7C01%7Cyguerra%40cookcountyhhs.org%7C74cbdfb597bb4f78587008d78dee6e59%7C3b922295e886417faaa84e4c4f069d82%7C0%7C1%7C637133927514874831&sdata=M3vjJXE%2Bd2oBHdbp9ZORkRjWhJX2NJI6s6grty7y4TA%3D&reserved=0).

Reviewer #1: No

Reviewer #2: No

Reviewer #3: No


[NOTE: If reviewer comments were submitted as an attachment file, they will be attached to this email and accessible via the submission site. Please log into your account, locate the manuscript record, and check for the action link "View Attachments". If this link does not appear, there are no attachment files to be viewed.]


While revising your submission, please upload your figure files to the Preflight Analysis and Conversion Engine (PACE) digital diagnostic tool, [https://pacev2.apexcovantage.com/](https://gcc01.safelinks.protection.outlook.com/?url=https%3A%2F%2Fpacev2.apexcovantage.com%2F&data=02%7C01%7Cyguerra%40cookcountyhhs.org%7C74cbdfb597bb4f78587008d78dee6e59%7C3b922295e886417faaa84e4c4f069d82%7C0%7C1%7C637133927514884826&sdata=kn0NlcVtYzHPgUIss%2Fyx32VmuFPgIt9yXflxqTiyqz8%3D&reserved=0). PACE helps ensure that figures meet PLOS requirements. To use PACE, you must first register as a user. Registration is free. Then, login and navigate to the UPLOAD tab, where you will find detailed instructions on how to use the tool. If you encounter any issues or have any questions when using PACE, please email us at [figures@plos.org](mailto:figures@plos.org). Please note that Supporting Information files do not need this step.
